# Novel (1S,3R)-RSL3-Encapsulated Polyunsaturated Fatty Acid Rich Liposomes Sensitise Multiple Myeloma Cells to Ferroptosis-Mediated Cell Death

**DOI:** 10.3390/ijms26146579

**Published:** 2025-07-09

**Authors:** Ali Habib, Rachel L. Mynott, Oliver G. Best, Isabella A. Revesz, Clive A. Prestidge, Craig T. Wallington-Gates

**Affiliations:** 1 College of Medicine and Public Health, Flinders University, Bedford Park, SA 5042, Australia; ali.habib@flinders.edu.au (A.H.); rachel.mynott@flinders.edu.au (R.L.M.); giles.best@flinders.edu.au (O.G.B.); 2 Clinical Health Sciences, University of South Australia, Adelaide, SA 5005, Australia; isabella.revesz@unisa.edu.au (I.A.R.); clive.prestidge@unisa.edu.au (C.A.P.); 3 School of Health, University of the Sunshine Coast, Sippy Downs, QLD 4556, Australia; 4 School of Medicine and Dentistry, Griffith University, Birtinya, QLD 4575, Australia; 5 Department of Haematology, Sunshine Coast University Hospital, Birtinya, QLD 4575, Australia

**Keywords:** cancer, haematology, multiple myeloma, ferroptosis, phospholipids, fatty acids, saturated, unsaturated, nanotechnology, nanoparticles, liposomes

## Abstract

Multiple myeloma (MM) is an incurable malignancy of plasma cells that accounts for 10% of all haematological malignancies diagnosed worldwide. The poor outcome of patients with MM highlights the ongoing need for novel treatment strategies. Ferroptosis is a recently characterised form of non-apoptotic programmed cell death. Phospholipids (PLs) containing polyunsaturated fatty acids (PUFAs) play a crucial role as ferroptosis substrates when oxidised to form toxic lipid reactive oxygen species (ROS). Using a range of scientific techniques, we demonstrate a strong correlation between the PL profile of MM and diffuse large B cell lymphoma (DLBCL) cells with their sensitivity to ferroptosis. Using this PL profiling, we manufacture liposomes that are themselves composed of PL-PUFA ferroptosis substrates relatively deficient in MM cells, with and without the GPX4 inhibitor, RSL3, for investigation of their ferroptosis-inducing potential. PL-PUFAs were more abundant in DLBCL than MM cell lines, consistent with greater ferroptosis sensitivity. In contrast, MM cells generally contained a significantly higher proportion of PLs containing monounsaturated fatty acids. Altering the lipid composition of MM cells through exogenous supplementation with PL-PUFAs induced ferroptosis-mediated cell death and further sensitised these cells to RSL3. Liposomes predominantly comprising PL-PUFAs were subsequently manufactured and loaded with RSL3. Uptake, cytotoxicity and lipid ROS studies demonstrated that these novel liposomes were readily taken up by MM cells. Those containing RSL3 were more effective at inducing ferroptosis than empty liposomes or free RSL3, resulting in IC_50_ values an average 7.1-fold to 14.5-fold lower than those for free RSL3, from the micromolar to nanomolar range. We provide a better understanding of the mechanisms associated with ferroptosis resistance of MM cells and suggest that strategies such as liposomal delivery of relatively deficient ferroptosis-inducing PL-PUFAs together with other targeted agents could harness ferroptosis for the personalised treatment of MM and other cancers.

## 1. Background

Multiple myeloma (MM) is the second most common haematological malignancy worldwide and is characterised by the clonal proliferation of plasma cells in the bone marrow [1,2]. Despite advances in treatment, MM is still considered incurable. Between 2016 and 2020, the 5-year survival rate for patients with MM in Australia was 59.5%, with 10,848 people living with the disease at the end of 2020 who were diagnosed between 2011 and 2020 [3]. Although survival rates among MM patients are predicted to improve with the advent of recent therapeutic advances [4,5], there remains an ongoing need for novel treatment approaches. This is particularly important for the significant proportion of patients who relapse or develop disease that is resistant to standard therapies.

The efficacy of many cancer therapies, including those used to treat MM patients, is dependent on their ability to induce apoptosis-mediated cell death. However, tumorigenesis and disease evolution are often associated with resistance to many of the current treatment options [6,7]. Relatively recently, an iron-dependent form of programmed cell death (PCD), termed ferroptosis, was described [8]. Ferroptosis is characterised by lipid peroxidation, which leads to breakdown of cell membranes and disruption of cellular homeostasis. Given that the intracellular signalling pathways involved in ferroptosis are distinct from other forms of PCD [8,9,10], induction of ferroptosis may represent a promising therapeutic strategy for targeting apoptosis-resistant cells with the potential to significantly improve outcomes for cancer patients, including those with MM.

Phospholipids (PLs) play crucial roles in many cellular processes and are the main constituent of cellular membranes [11]. The peroxidation of certain PLs, specifically glycerophospholipids, is also crucial for the initiation and propagation of ferroptosis [11,12,13,14]. PLs consist of a polar phosphate head region and two nonpolar fatty acid chains linked by a glycerol backbone [11,15,16]. PLs are characterised according to the head group as this largely dictates the chemical properties, structure, function, and membrane localisation of the lipid [11,15,16]. The fatty acids (FAs) that make up the acyl chains within PLs are carboxylic acids with an aliphatic chain. FAs can be categorised into three distinct classes: saturated fatty acids (SFAs), monounsaturated fatty acids (MUFAs) and polyunsaturated fatty acids (PUFAs). Unlike SFA and MUFA, PUFAs are readily oxidised and represent important substrates for lipid peroxidation [12].

Phosphatidylethanolamine (PE) lipids predominantly comprise PUFAs and are abundant in the inner leaflet of the plasma membrane [12]. The susceptibility of PE lipids to oxidation means they are important substrates for ferroptosis [12], although other PLs, including phosphatidylcholine (PC) and phosphatidylserine (PS), are also readily oxidised [12,14,17]. In addition to the class of PL, the degree of unsaturation also affects how readily lipids are oxidised [12].

The lipid peroxidation that occurs during ferroptosis is associated with the formation of potent oxidising agents, particularly hydroxyl radicals, via the Fenton reaction [8]. Hydroxyl radicals remove a bis-allylic hydrogen atom from PL-PUFAs, forming carbon-centred PLs, which react with oxygen molecules to form highly potent PL peroxyl radicals that trigger ferroptotic cell death [14]. Regulation of lipid peroxidation is complex and involves several key molecules, including the antioxidant glutathione peroxidase 4 (GPX4) [9,18]. GPX4 is a selenoprotein which converts toxic lipid peroxides into neutral alcohols and, in doing so, inhibits lipid peroxidation and ferroptosis [19,20]. Given the role of lipid peroxidation in ferroptosis, lipid-based nanostructures, e.g., liposomes, may represent a promising means of delivering therapeutic agents and ferroptosis substrates to cells that may otherwise be relatively insensitive to this form of cell death.

Liposomes are extremely versatile spherical vesicles that primarily consist of lipids and range in size from 30 nm to a few micrometres [21]. The lipid-rich nature of these nanoparticles enables hydrophilic agents to be encapsulated within the aqueous core, which is surrounded by the hydrophobic lipid bilayer. Although small vesicles such as liposomes are typically taken up by cells via endocytosis, uptake can be modified and improved by controlling their lipid composition [22]. The versatility of liposomes lies in the ability to modify their surface with polymers, antibodies and proteins. Adding components or surface functionalising liposomes may significantly expand their possible therapeutic applications by enabling the delivery of macromolecular drugs or genetic therapies (e.g., siRNA) in a highly targeted manner.

The use of nanotechnologies aimed at inducing ferroptosis in cancer cells has grown exponentially in recent years, with studies in non-small lung cancer, breast cancer, colon cancer, colorectal cancer, ovarian cancer and skin cancers [23,24,25,26,27,28]. Many of these studies focused on nanotechnologies other than liposomes, capable of delivering iron to the cells. However, two recent studies in which both ferroptosis- and apoptosis-inducing compounds were encapsulated within liposomes demonstrate the potential of these nanoparticles as a means of overcoming drug resistance [29,30].

MM cells are inherently less sensitive to ferroptosis-inducing compounds than diffuse large B cell lymphoma (DLBCL) cells, as shown in the study by Yang et al., who tested the efficacy of the cysteine antiporter X_C_^−^ inhibitor, erastin [9]. Despite their relative insensitivity to erastin, it is apparent that MM cells can undergo ferroptosis [31,32]. However, realising the potential of ferroptosis as a novel approach for the treatment of haematological malignancies [18] requires a better understanding of the mechanisms related to ferroptosis sensitivity in these cancers.

In the current study, we performed lipidomic analyses which identified a strong association between cellular lipid composition and the ferroptosis sensitivity of MM and DLBCL cells. Liposome nanoparticles were then manufactured from ferroptosis-inducing PL-PUFAs, identified in the lipidomic analyses to be relatively deficient in MM cells, and loaded with the GPX4 inhibitor, RSL3. Functional studies demonstrate that the liposomes were rapidly taken up by MM cells and that the delivery of PL-PUFAs as ferroptosis substrates and RSL3 in a liposomal formulation was significantly more potent than exogeneous PL-PUFAs in combination with free RSL3 at inducing ferroptosis-mediated cell death.

The findings of this study highlight the importance of cellular lipid composition in relation to ferroptosis sensitivity and provide a proof-of-principle that liposomal nanoparticles, themselves composed of PL-PUFA ferroptosis substrates and containing other targeted therapeutics, may represent an effective means of inducing ferroptosis in MM and other cancer cells that are less sensitive to this form of programmed cell death. Moreover, this research suggests there is enormous potential for developing precision or personalised lipid nanoparticle therapeutics for induction of ferroptosis.

## 2. Results

### 2.1. MM Cells Are Less Sensitive to the GPX4 Inhibitor, RSL3, than DLBCL Cells

**Figure 1:**
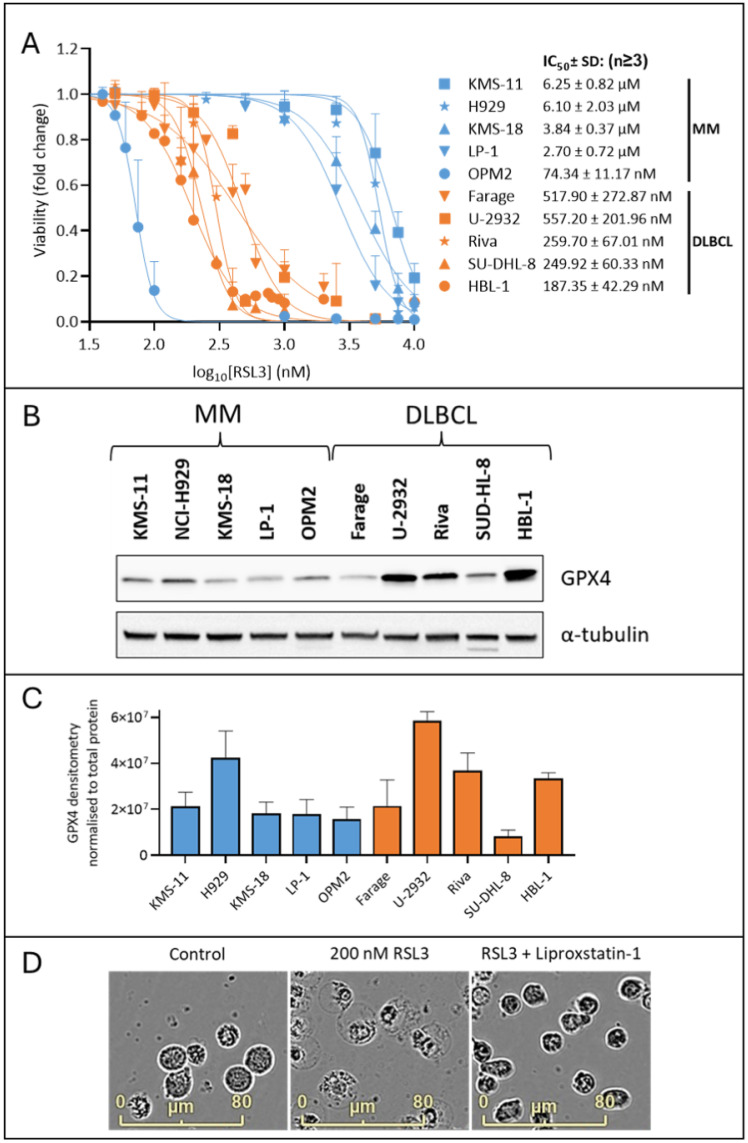
MM cells are generally less sensitive to RSL3 induced cell death compared to DLBCL cells. (**A**) Cell viability was assessed by annexin V/PI staining and flow cytometry in MM and DLBCL cell lines treated with RSL3 for 24 h. Dual annexin V/PI negative cells were considered viable. The mean ± standard deviation of duplicate measurements are shown from at least 3 independent experiments. (**B**) Western blot for GPX4 expression in untreated MM and DLBCL cells. Alpha tubulin expression was assessed as a loading control. (**C**) Western blot densitometry data are mean (normalised to total protein) ± standard deviation from a minimum of three independent experiments. (**D**) Untreated OPM-2 cells, OPM-2 cells cultured with 200 nM RSL3, and OPM-2 cells cultured with RSL3 200 nM and 2 µM liproxstatin-1, for 24 h. Images were captured at 20× magnification using an IncuCyte^®^ S3 Live-Cell Analysis System and v2023A software. MM, multiple myeloma; DLBCL, diffuse large B cell lymphoma, GPX4, glutathione peroxidase 4; RSL3, (1S,3R)-RSL3.

The glutathione peroxidase 4 (GPX4) enzyme catalyses the reduction of PL hydroperoxides into their corresponding alcohols, which decreases levels of lipid ROS and inhibits ferroptosis [33]. Indirect inhibition of GPX4 with erastin has been shown to induce ferroptosis in DLBCL, but not MM, cell lines [9]. We demonstrated similar effects in MM and DLBCL cells using RSL3, a small molecule inhibitor that directly inhibits GPX4 by binding to the catalytic selenocysteine residue of the enzyme (Figure 1A). Cell death was observed in all the DLBCL cell lines with an average IC_50_ value of 354.41 ± 170.04 nM. With the exception of OPM-2 cells, the MM lines were much less sensitive to RSL3, with and average IC_50_ value of 4722.50 ± 1741.65 nM (excluding the OPM-2 MM cell line) (Figure 1A). Western blot analysis of whole cell lysates from the MM and DLBCL cell lines suggested differences in the sensitivity to RSL3 between the lines were not due to varying expression of its target, GPX4 (Figure 1B,C). Ferroptosis is associated with characteristic changes in cell morphology, including a “ballooning” phenotype due to an enlarged cytoplasm. Using an IncuCyte^®^ S3 Live-Cell Analysis System, we observed morphological changes that are consistent with ferroptosis in both the MM and DLBCL cells, with the RSL3 sensitive OPM-2 cell line displaying ferroptotic morphology when cultured with 200 nM RSL3 for 24 h (Figure 1D). The effects of RSL3 on cell morphology were inhibited by the synthetic antioxidant, liproxstatin-1, which supports the assertion that the changes observed were associated with ferroptosis.

To further demonstrate that ferroptosis is the major mechanism of cell death in MM cell lines cultured with RSL3, MM cells were cultured with bortezomib that is known to induce apoptosis in MM cells [34] with and without the pan-caspase inhibitor Z-VAD-FMK or liproxstatin-1, and compared to similar cell cultures using RSL3 (Supplementary Figure S1A). Z-VAD-FMK was able to prevent the majority of cell death induced by bortezomib but not with RSL3, which was near completely prevented by liproxstatin-1. Moreover, we observed morphological changes that are consistent with either apoptosis or ferroptosis when OPM2 cells were cultured with either bortezomib or RSL3, respectively (Supplementary Figure S1B). The possibility that another form of cell death, known as necroptosis, was involved in the effects of RSL3 against MM cells was investigated using the receptor-interacting serine/threonine-protein kinase 1 (RIPK1) inhibitor necrostatin-1s (Nec-1s). In necroptosis, RIPK1 and RIPK3 form a necrosome complex, resulting in a signalling cascade that ends in phosphorylation of mixed lineage kinase domain-like protein (MLKL) and execution of necroptosis [35]. Nec-1s did not prevent RSL3-induced cell death or lipid oxidation, indicating that necroptosis does not play a role in the toxicity of RSL3 against MM cells (Supplementary Figure S2A,B). Furthermore, Western blotting showed that none of the MM cell lines express RIPK3, which is a protein that is crucial for activation of necroptosis (Supplementary Figure S2C).

### 2.2. MM Cells Generally Contain Higher Proportions of PL-MUFA than DLBCL Cell Lines

Studies show that PLs containing PUFAs represent important substrates for ferroptosis, [12] and that the peroxidation of these lipids is a crucial step in ferroptosis [36]. To investigate this, the PL profiles of the MM (*n* = 5) and DLBCL cell lines (*n* = 5) were analysed by liquid chromatography–mass spectrometry (LC-MS).

**Figure 2:**
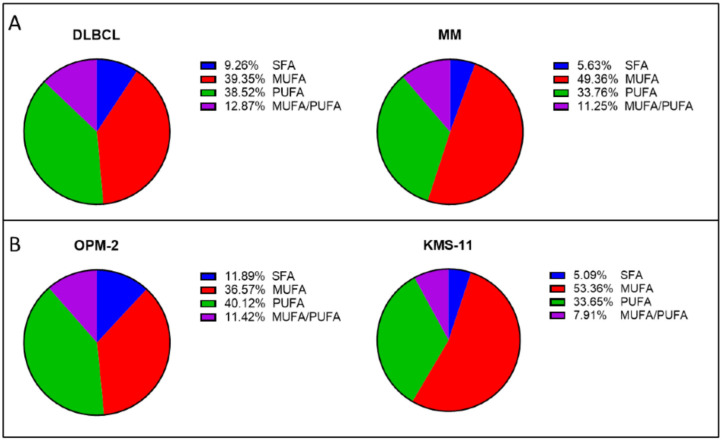
Distinct differences in the phospholipid composition of MM and DLBCL cells. (**A**) Analysis of data pooled from the MM (excluding ferroptosis-sensitive OPM-2) and DLBCL lines showing the proportions of each PL acyl chain. Proportions were calculated as the peak area of each acyl chain relative to the total PL peak area by LC-MS. Data are the mean from a minimum of 4 biological replicates per cell line. (**B**) PL composition in the OPM-2 and KMS-11 MM lines showing the proportions of each PL acyl chain. Data are the mean from a minimum of 12 biological replicates per cell line. All statistical analyses (see text) were performed using Student’s *t*-test using a minimum of 4 biological replicates. MM, multiple myeloma; DLBCL, diffuse large B cell lymphoma; MUFA, monounsaturated fatty acid; PUFA, polyunsaturated fatty acid; SFA, saturated fatty acid.

To examine the acyl chains within the PLs in more detail, the PLs were grouped as follows: (1) PLs with completely saturated acyl chains (SFA), (2) PLs containing a combination of SFA and MUFA (MUFA), (3) PLs containing both MUFA and PUFA (MUFA/PUFA), or (4) PLs containing a combination of SFA and PUFA (PUFA). Due to their low abundance, phospholipids containing SFA/MUFA or MUFA/MUFA were grouped together and those containing SFA/PUFA or PUFA/PUFA were also grouped together. PLs typically contain a SFA in the *sn1* position, whereas the *sn2* position can contain SFA, MUFA or PUFA [37]. Analysis of the data revealed that MM cell lines had a higher proportion of PLs containing MUFAs than the DLBCL cell lines (Figure 2A). In contrast, DLBCL cell lines contained a higher proportion of PLs containing PUFAs than MUFAs (Figure 2A). Studies suggest that MUFAs play a role in protecting cells against ferroptosis by preventing accumulation of toxic lipid ROS and reducing the amount of PUFAs that are incorporated into PLs [12,38,39].

The most notable difference between the two cancer types appears to be in the proportions of PLs containing PUFA or MUFA (Figure 2A). The DLBCL cell lines were found to contain significantly (*p* < 0.01) higher levels of PL-PUFA (38.52% ± 2.72% of the total phospholipidome) in comparison to the MM cell lines (33.76% ± 6.20%) (Figure 2A). In contrast, the MM cell lines had significantly (*p* < 0.0001) higher levels of PL-MUFA, which constituted up to 49.36% ± 7.58% of their total PL content, compared to 39.35 ± 6.62% in the DLBCL cells (Figure 2A). There was also a significant (*p* < 0.001) difference between DLBCL and MM lines in the levels of PLs containing SFA only, however SFA levels were under 10% in both cancers (Figure 2A). There was no significant difference between the MUFA/PUFA groups when comparing MM and DLBCL (Figure 2A). In addition to distinct differences between the two cancers, differences in the PL composition between each of the cell lines were also observed (Supplementary Figure S3).

Next, we compared the phospholipidome of the ferroptosis sensitive and resistant MM cell lines, OPM-2 and KMS-11, respectively (Figure 2B). A higher proportion (40.12% ± 8.44%) of PUFA were identified in the OPM-2 cells compared to MUFA (36.57 ± 11.91%) (Figure 2B). In contrast, the lipid profile of KMS-11 cells was almost the reverse, with the largest proportion of lipids identified as MUFAs (53.36 ± 4.20%), with a significantly (*p* < 0.0001) smaller proportion of PUFAs (33.65 ± 2.62%) (Figure 2B). There was also a statistically significant difference in MUFA (*p* < 0.001) and PUFA (*p* < 0.05) between the two cell lines (Figure 2B). Interestingly, the OPM-2 cell line had significantly greater proportions of SFA (*p* < 0.01) and MUFA/PUFA (*p* < 0.001) than the KMS-11 cell line (Figure 2B). Some studies suggest that SFA plays a role in promoting ferroptosis induction [40,41], while others suggest that they play a protective role [42], indicating that the effects of SFAs may be context dependent. The role of PLs containing both MUFA and PUFA has yet to be fully elucidated in the literature but may influence ferroptosis sensitivity.

### 2.3. Exogenous PL-PUFA Induces Ferroptosis in MM Cells Proportional to the Degree of Acyl Chain Saturation

**Figure 3:**
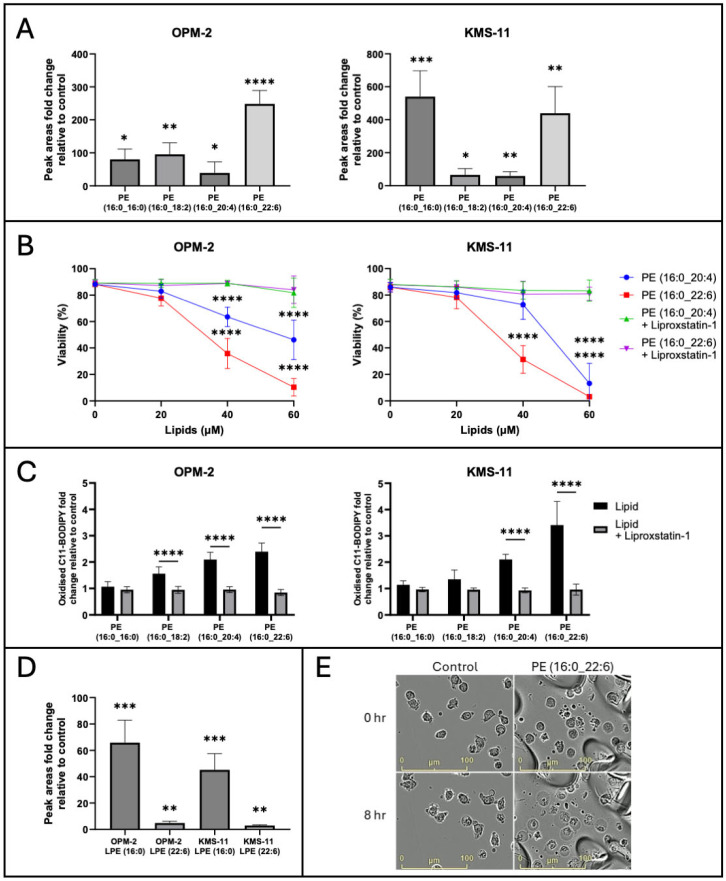
Ferroptosis induced by exogenous PL-PUFA correlates with the degree of acyl chain saturation. (**A**) OPM-2 and KMS-11 cells were cultured with 20 µM of the lipids indicated. Lipid uptake was assessed by LC-MS. Data are presented as mean fold changes relative to unmanipulated cell lines ± standard deviation from 3 independent experiments and statistical analyses performed using Student’s *t*-test for statistical analysis (* *p* < 0.05, ** *p* < 0.01, *** *p* < 0.001, **** *p* < 0.0001). (**B**) OPM-2 and KMS-11 MM cells were cultured with the indicated concentrations of the PE lipids. Cell viability was assessed by flow cytometry following staining with annexin V and PI. Dual annexin V/PI negative cells were considered viable. Data are the mean ± standard deviation of duplicate measurements from 3 independent experiments using two-way ANOVA for statistical analysis (**** *p* < 0.0001). (**C**) OPM-2 and KMS-11 MM cells were cultured with 40 µM of the PE lipids, ± liproxstatin-1, as indicated. Lipid ROS levels were assessed by flow cytometry in cells stained with C11 BODIPY. Data are the mean ± standard deviation of duplicate measurements from 3 independent experiments using two-way ANOVA for statistical analysis (**** *p* < 0.0001). (**D**) OPM-2 and KMS-11 MM cells were cultured with 20 µM PE (16:0_22:6) for 4 h. LPE levels were determined by LC-MS. Data are presented as mean fold changes ± standard deviation from 3 independent experiments and statistical analyses performed using Student’s *t*-test for statistical analysis (** *p* < 0.01, *** *p* < 0.0001). (**E**) OPM-2 cells were cultured with or without 60 µM PE (16:0_22:6). Images were acquired at the 0 and 8 h time points using an IncuCyte S3 live cell analysis system at 20× magnification. LPE, lysophosphatidylethanolamine; PE, phosphatidylethanolamine.

Given that PUFA but not MUFA are readily oxidised leading to accumulation of lipid ROS and ferroptosis [12], we hypothesised that the ratio of PL-PUFA to PL-MUFA may dictate the sensitivity of MM cells to ferroptosis. OPM-2 and KMS-11 MM cells were cultured with four PE lipids with different degrees of acyl chain saturation, that were identified from the lipidomic analysis and from the literature (Figure 3B, Supplementary Figure S3) [38,43]. Both cell lines demonstrated the capacity to take up the four lipids studied, as demonstrated by the significant increase in the levels of these lipids in cell lysates analysed by LC-MS (Figure 3A). However, uptake of the lipids varied between the two cell lines. The addition of PE (16:0_16:0) resulted in an 80.1 ± 31.4-fold increase in the intracellular levels of PL in the OPM-2 cell line, while a fold change increase of 540.3 ± 156.9 was observed in KMS-11 cells (Figure 3A). Uptake of PE (16:0_18:2) was observed in both cell lines, with a 95.4 ± 34.8-fold increase observed in OPM-2 cells and a 65.5 ± 38.1-fold increase in KMS-11 cells (Figure 3B). The uptake of PE (16:0_20:4) was also observed in the two lines, with fold changes of 39.3 ± 33.3 and 58.8 ± 26.0 in the OPM-2 and KMS-11 cells, respectively. Following the addition of PE (16:0_22:6), we observed a 248.2 ± 41.0-fold increase in this lipid in OPM-2 cells, compared to a 439.3 ± 161.4-fold change in KMS-11 cells (Figure 3A).

PE (16:0_20:4) and PE (16:0_22:6) induced cell death of both cell lines, with IC_50_ values of 53.61 ± 5.62 µM and 33.99 ± 15.34 µM for OPM-2 cells, and 54.19 ± 2.51 µM and 37.33 ± 9.03 µM for KMS-11 cells, respectively (Figure 3B). The cytotoxic effects of these lipids in both cell lines were inhibited by the synthetic antioxidant, liproxstatin-1, suggesting the cell death observed was due to ferroptosis (Figure 3B). In contrast, no cytotoxic effects induced by PE (16:0_16:0) or PE (16:0_18:2) were observed.

Treatment of OPM-2 and KMS-11 cells with PL-PUFA increased lipid ROS levels, as demonstrated by changes in the levels of oxidised C11 BODIPY; statistically significant fold changes of >2 relative to unmanipulated cells were observed in both OPM-2 and KMS-11 cell lines following treatment with 40 µM PE (16:0_20:4) or PE (16:0_22:6) (Figure 3C). A statistically significant increase in oxidised C11 BODIPY, without a decrease in cell viability, was also observed in OPM-2 when cultured with PE (16:0_18:2). The KMS-11 cells displayed a smaller, non-significant, fold change increase in oxidised C11 BODIPY following treatment with PE (16:0_18:2) (Figure 3C). Treatment with PE (16:0_16:0) had no significant effect on lipid ROS or cell viability in either cell line (Figure 3C). In all cases, the increases in lipid ROS levels were prevented by liproxstatin-1, consistent with induction of ferroptosis (Figure 3C).

Interestingly, both OPM-2 and KMS-11 cells were found to contain significantly higher levels of the lysophospholipids, lysophosphatidylethanolamine (LPE) (16:0) and LPE (22:6), following treatment with PE (16:0_22:6) (Figure 3D). Lysophospholipids are characterised by a polar head group and a singular carbon chain and are typically a minor component of the total lipid composition of cells [44]. However, studies have shown that levels of lysophospholipids significantly increase during ferroptosis, with a concomitant decrease in the corresponding PUFA-containing PL [9,45]. In OPM-2 cells, LPE (16:0) and LPE (22:6) increased 65.9 ± 17.0 and 4.9 ± 1.3-fold, respectively, while in KMS-11 cells, the same lipids increased by 45.2 ± 12.4 and 2.9 ± 0.6-fold, respectively (Figure 3D). No significant change in the levels of these lysophospholipids was observed in either cell line following culture with the other lipids. These findings indicate that PE (16:0_22:6) may be consumed during ferroptosis, resulting in the formation of the corresponding lysophospholipids.

Next, we examined the effects of the lipids on the morphology of MM cells. OPM-2 cells were cultured with PE (16:0_22:6) and images captured over a 24 h time frame using an IncuCyte S3 instrument (Figure 3E). The images show that the cells underwent morphological changes characteristic of ferroptosis, including cytoplasmic “ballooning”, in response to addition of the lipid (Figure 3E) [46]. These changes were evident in cells treated with the lipid from 8 h onwards; an additional movie file shows this in more detail (see Supplementary File S1).

### 2.4. Exogenous PL-PUFA and RSL3 Synergise, Inducing Ferroptosis-Mediated Cell Death

**Figure 4:**
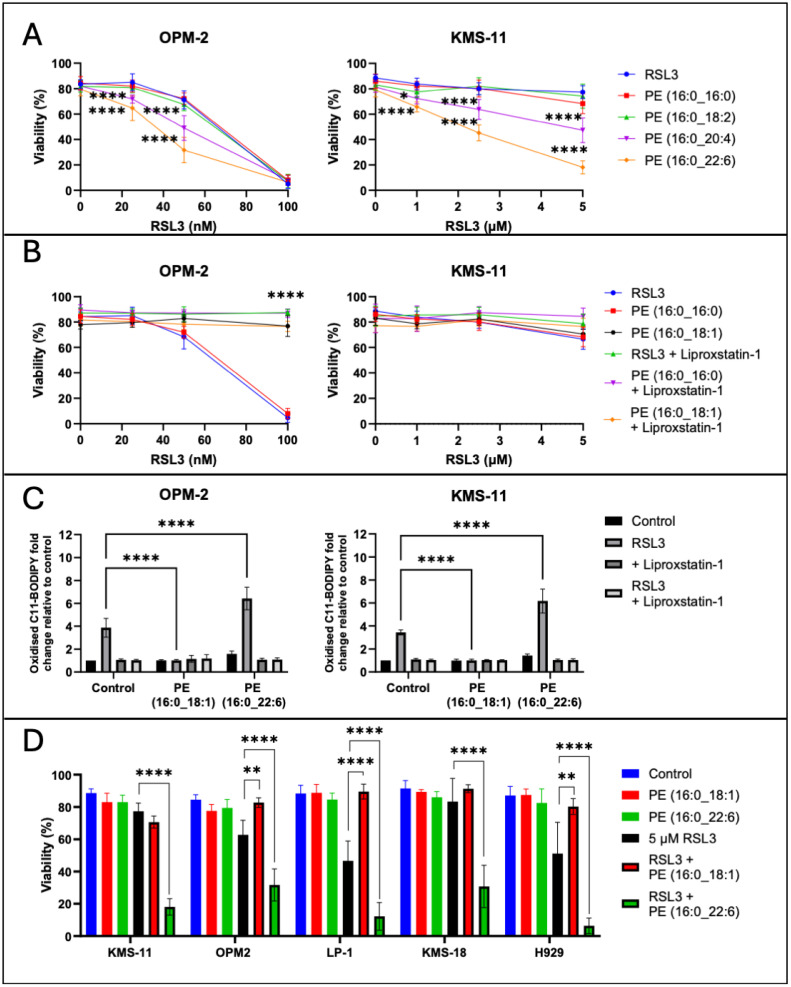
PL-PUFA and RSL3 induce synergistic, ferroptosis-mediated cell death while PL-MUFA protect cells from ferroptosis. (**A**) OPM-2 and KMS-11 cells were cultured with 20 μM PE (16:0_20:4) or PE (16:0_22:6) and the concentrations of RSL3 indicated. Cell viability was assessed using annexin V/PI staining and flow cytometry. Dual annexin V/PI negative cells were considered viable. (**B**) OPM-2 and KMS-11 cells were cultured with 20 μM PE (16:0_16:0) or PE (16:0_18:1) and RSL3. Cell viability was assessed by flow cytometry using annexin V/PI and flow cytometry. (**C**) OPM-2 and KMS-11 were cultured with PE (16:0_22:6) or PE (16:0_18:1) and RSL3 ± liproxstatin-1. Lipid ROS levels were assessed by flow cytometry in cells stained with C11 BODIPY. Data are fold change relative to untreated control. (**D**) MM cell lines were cultured with 1 μM (KMS-11, LP-1, KMS-18, H929) or 50 nM (OPM-2) RSL3 for 24 h, with or without 20 μM PE (16:0_18:1) or PE (16:0_22:6). Cell viability was assessed by flow cytometry following staining with annexin V and PI. All data are the mean ± standard deviation of duplicate measurements from 3 independent experiments using two-way ANOVA for statistical analysis (* *p* < 0.05, ** *p* < 0.01, **** *p* < 0.0001).

Having determined that addition of exogenous PL-PUFA alters the lipidome of MM cells (Figure 3A) and can induce ferroptotic cell death (Figure 3B), we hypothesised that altering the lipid composition of MM cells may also sensitise these cells to ferroptosis induced by the GPX4 inhibitor, RSL3. Combinations of RSL3 and PE (16:0_20:4) or PE (16:0_22:6) were synergistic in OPM-2 MM cells; in combination with PE (16:0_20:4) or PE (16:0_22:6) the IC_50_ values for RSL3 were 43.59 ± 5.24 nM and 34.11 ± 6.54 nM, respectively, compared to an IC_50_ of 74.34 ± 11.17 nM for RSL3 alone (Figure 1A and Figure 4A). In the KMS-11 cells PE (16:0_20:4) and PE (16:0_22:6) significantly increased the sensitivity of the cells to RSL3; IC_50_ values for RSL3 in combination with the lipids were 4.86 ± 2.05 µM and 2.13 ± 0.69 µM, respectively, while the IC_50_ value for RSL3 alone was 6.25 ± 0.82 µM (Figure 1A and Figure 4A). Synergistic cell death with RSL3 was observed for both PE (16:0_20:4) and PE (16:0_22:6), with fractional products of −0.3 and −0.75 for the two lipids, respectively (Figure 4A, fractional products of <−0.1 are indicative of synergy [47]). In both OPM-2 and KMS-11 cells, addition of liproxstatin-1 prevented the cell death induced by combinations of RSL3 and the lipids, consistent with a ferroptosis-mediated mechanism of cell death (Supplementary Figure S4A).

While PL-PUFA are substrates for ferroptosis, high proportions of PL-MUFA are thought to protect cells from this form of programmed cell death [12,39]. To explore this possibility in the context of MM, OPM-2 and KMS-11 cell lines were treated with RSL3 in combination with either PE (16:0_16:0) or PE (16:0_18:1) (Figure 4B). Addition of PE (16:0_18:1) to OPM-2 cells inhibited both ferroptosis-mediated cell death and the accumulation of lipid ROS in response to RSL3, similar to the effects observed with liproxstatin-1 (Figure 4B,C). In contrast, PE (16:0_16:0) had no significant effect on the response of OPM-2 cells to RSL3 (Figure 4B). Given 5 µM RSL3 did not induce cell death in KMS-11 cells, the addition of either PE (16:0_16:0) or PE (16:0_18:1) had no effect on the sensitivity of these cells to RSL3 (Figure 4B).

Combining RSL3 and PE (16:0_22:6) augmented lipid ROS generation in both the OPM-2 and KMS-11 cell lines (Figure 4C), suggesting that increasing cellular PL-PUFA content increases the availability of substrates for lipid ROS generation. The increase in lipid ROS generation when PE (16:0_22:6) was added to RSL3 was also inhibited by liproxstatin-1 in both cell lines (Figure 4C and Supplementary Figure S4B). Furthermore, the increase in lipid ROS levels induced by RSL3 was prevented by PE (16:0_18:1) in both cell lines (Figure 4C).

Interestingly, LC-MS analysis did not show a significant increase in PE (16:0_18:1) in cells treated with this lipid. However, it appeared that levels of other oleic acid (18:1)-containing PLs were increased throughout the phospholipidome of both OPM-2 and KMS-11, while the proportion of PL-PUFA decreased (Supplementary Figure S5). Cells can metabolise exogenous PLs, such as PE (16:0_18:1), breaking them down into free fatty acids and thus allowing them to be incorporated into other PLs [38,48]. This may explain why oleic acid, but not specifically PE (16:0_18:1) levels increased in the treated samples, however further experiments would be required to determine the fate of this lipid.

To study the effects of specific PE lipids on MM cells more broadly, three additional MM cell lines were treated with RSL3 and either PE-PUFA or PE-MUFA (Figure 4D). Consistent with our findings from the OPM-2 and KMS-11 cell lines, treatment of LP-1 and H929 MM cell lines with RSL3 in combination with PE (16:0_18:1), led to a significant reduction in cell death (Figure 4D). However, this effect was not observed in the KMS-18 cell line. Also consistent with our earlier findings, we observed synergy between RSL3 and PE (16:0_22:6) in all five of the MM cell lines, suggesting this is not a cell line dependent effect (Figure 4D).

### 2.5. Induction of Ferroptosis in MM Cells by PL-PUFA-Rich Liposomes

The lipidomic data presented was used to inform the manufacture of novel liposomes, predominantly composed of PE (16:0_22:6) (98%), which induced ferroptosis-mediated cell death when added exogenously to the MM cells (Figure 3B). The remaining 2% was a pegylated saturated fatty acid, DSPE-PEG2000, which was added to stabilise the liposomes [49]. The mean diameter of the synthesised liposomes was 135 nm with a polydispersity index (PDI) of 0.06 and neutral charge of −2 mV. PDI represents the size distribution of liposomes, with values under 0.30 suggesting nanoparticles of uniform size. To assess their stability, liposomes were stored for 2 weeks at 4 °C after synthesis; the mean diameter of the liposomes after 2 weeks was 131 nm with a PDI of 0.18, confirming they were stable within this time frame.

**Figure 5:**
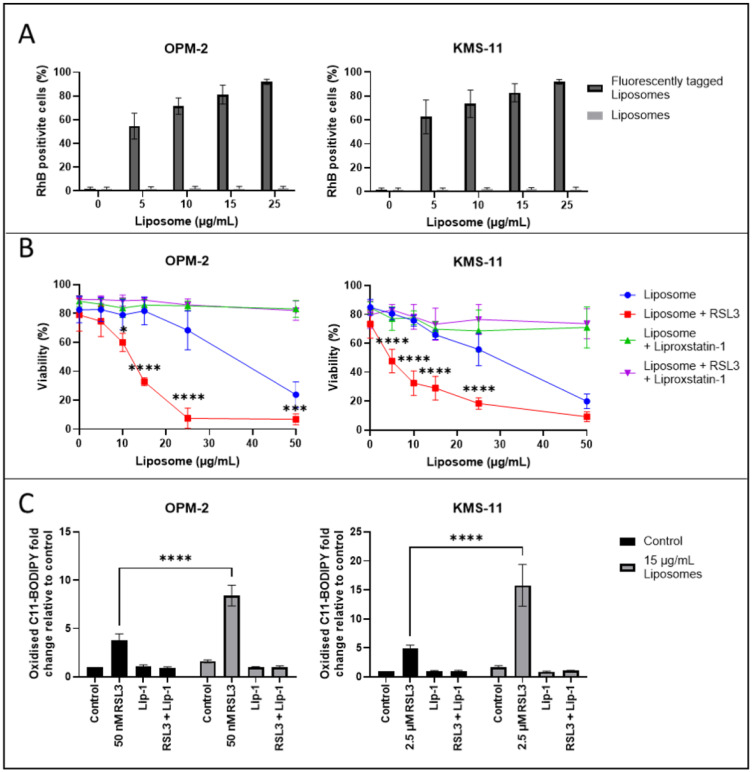
Liposomes composed of PE (16:0_22:6) combined with free RSL3 induce synergistic ferroptosis-mediated cell death of MM cells. (**A**) OPM-2 and KMS-11 cells were cultured for 24 h with increasing concentrations of liposomes, which did or did not contain PE (18:1) (lissamine rhodamine B sulfonyl)-conjugated lipid at a 1 in 1000 dilution. The percentage of cells containing the fluorescently tagged lipid was assessed by flow cytometry. (**B**) OPM-2 and KMS-11 cells were cultured with increasing concentrations of liposomes, with or without 50 nM (OPM-2) or 2.5 μM (KMS-11) free RSL3 with or without liproxstatin-1. Cell viability was assessed using annexin V/PI staining and flow cytometry. (**C**) OPM-2 and KMS-11 cells were cultured with 15 μg/mL liposomes, plus 50 nM or 2.5 μM RSL3, respectively, ± liproxstatin-1. Lipid ROS levels were assessed by flow cytometry in cells stained with C11 BODIPY. All Data are the mean ± standard deviation of duplicate measurements from 3 independent experiments using two-way ANOVA for statistical analyses (*** *p* < 0.001, **** *p* < 0.0001).

Initially, liposomes, which included the fluorescently tagged lipid PE (18:1) (lissamine rhodamine B sulfonyl)-conjugated at a 1 in 1000 dilution, were synthesised to enable their cellular uptake to be assessed. OPM-2 and KMS-11 cells were cultured with varying amounts of the liposomes for 24 h and levels of the fluorescently tagged lipid in the cells were assessed by flow cytometry. Liposome uptake was similar in both the OPM-2 and the KMS-11 cells, with >80% of the cells analysed identified as containing the fluorescent lipid after the addition of 15 μg/mL of the liposomes to cell cultures for 24 h (Figure 5A).

Next, varying amounts of the liposomes were added to OPM-2 and KMS-11 MM cells for 24 h, with dose dependent cell death observed in both lines (Figure 5B). The IC_50_ values for the liposomes against the OPM-2 and KMS-11 cells were 36.80 ± 14.53 μg/mL and 33.28 ± 4.89 μg/mL, respectively (Figure 5B). A concomitant increase in levels of lipid ROS was also observed in cells treated with the liposomes, with a 1.6 ± 0.2-fold increase in the OPM-2 and a 1.7 ± 0.3-fold increase in the KMS-11 cells in response to a 15 μg/mL dose of the liposomes (Figure 5C). Addition of liproxstatin-1 prevented both the cell death and lipid ROS accumulation induced by the liposomes (Figure 5B,C).

Consistent with the results obtained using exogenous lipids and RSL3, addition of free RSL3 in combination with the liposomes significantly increased the cytotoxic effects of the nanoparticles. In combination with 50 nM or 2.5 μM RSL3, the IC_50_ values for the liposomes against the OPM-2 and KMS-11 cells were reduced to 10.61 ± 2.91 μg/mL and 8.44 ± 2.73 μg/mL, respectively (Figure 5B). Synergy between 15 μg/mL of the liposomes and RSL3 was confirmed, with fractional products of −0.54 for the OPM-2 cell line and −0.33 for the KMS-11 cell line (Figure 5B). RSL3 also significantly increased the levels of ROS induced by the liposomes in both cell lines; 15 μg/mL of liposomes in combination with RSL3 resulted in an 8.4 ± 1.1-fold increase in lipid ROS in the OPM-2 cells and a 15.8 ± 3.6-fold increase in the KMS-11 cells (Figure 5C).

### 2.6. PL-PUFA-Rich Liposomes Containing RSL3 Induce Ferroptosis-Mediated Cell Death of MM Cells

**Figure 6:**
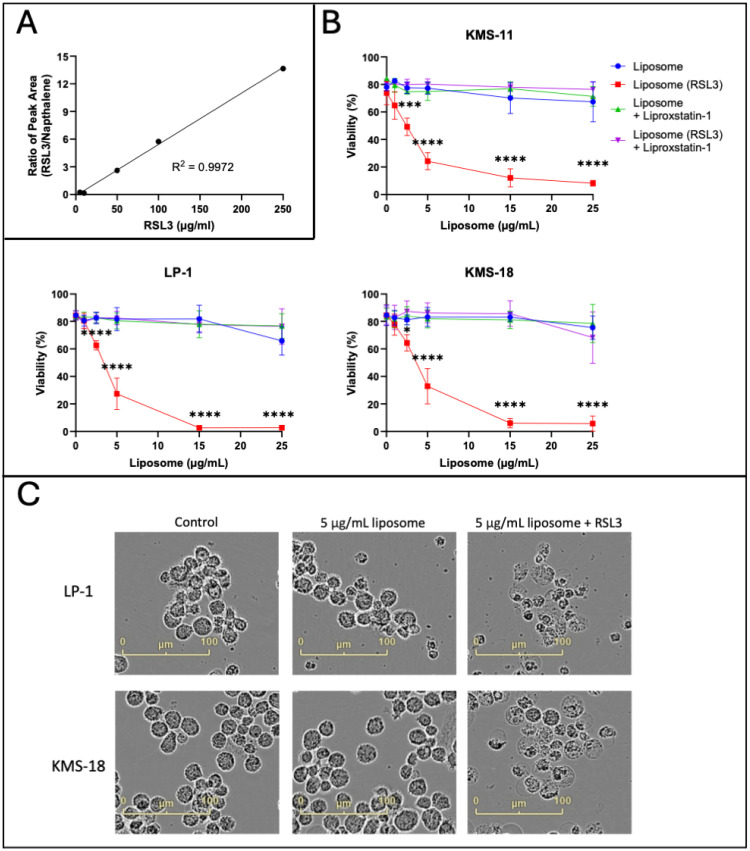
Liposomes encapsulating RSL3 are highly effective at inducing ferroptosis-mediated death of MM cells. (**A**) Representative standard curve generated for assessing RSL3 incorporation efficiency into liposomes, generated using peak areas from HPLC. (**B**) KMS-11, LP-1 and KMS-18 cells were cultured with increasing concentrations of liposomes that either did or did not contain RSL3 and with or without addition of free liproxstatin-1. Cell viability was assessed using annexin V/PI staining and flow cytometry. Data are the mean ± standard deviation of duplicate measurements from 3 independent experiments using two-way ANOVA for statistical analyses (*** *p* < 0.001, **** *p* < 0.0001). (**C**) MM cells were cultured for 24 h with 5 μg/mL liposomes that either did or did not contain RSL3. Images were acquired at 24 h using an IncuCyte S3 live cell analysis system at 20× magnification.

Next, RSL3 was encapsulated within the liposomes tested in Figure 5. In total, 50 μg/mL (113.41 µM) RSL3 was incorporated into the liposomes, corresponding to ~5% of the total liposome mass at assembly. RSL3 is a lipophilic molecule, which means it is likely to localise to the lipid bilayer of the liposomes. The mean diameter of the synthesised liposomes containing RSL3 was 135 nm with a PDI of 0.24 and a near neutral charge (zeta potential of −2 mV). A HPLC method for quantifying the concentration of RSL3 within the liposomes was developed based on the manufacturer’s guidelines for RSL3 purification and assessment (personal communication to A. Habib from Selleck Chemicals LLC). Increasing concentrations of RSL3 were prepared to generate standard curves (Figure 6A). RSL3 encapsulation efficiency was assessed using this methodology and was determined to be approximately 67%.

KMS-11, LP-1 and KMS-18 MM cell lines were cultured for 24 h with increasing concentrations of liposomes, which did or did not contain RSL3. The IC_50_ values for the RSL3-encapsulated liposomes in the aforementioned cell lines were 3.80 ± 0.67 μg/mL, 3.35 ± 0.70 μg/mL and 4.05 ± 1.51 μg/mL, respectively, which are equivalent to RSL3 concentrations of 430.39 ± 76.13 nM (KMS-11), 379.84 ± 79.25 nM (LP-1) and 459.47 ± 171.52 nM (KMS-18) (Figure 6B). In comparison, the IC_50_ values for free RSL3 against these lines were 6.25 ± 0.82 µM, 2.70 ± 0.72 µM, and 3.84 ± 0.37 µM, respectively (Figure 1A), consistent with an average 7.1-fold to 14.5-fold decrease in the IC_50_s by the RSL3-encapsulated liposomes from the micromolar to the nanomolar range. Morphological changes consistent with ferroptosis were observed in the MM cell lines after 24 h of treatment with the RSL3-containing liposomes (Figure 6C) and both the morphological changes and cell death were prevented by co-administration of liproxstatin-1 (Supplementary Figure S6 and Figure 6B). Additionally, increases in lipid ROS were also observed in the MM cells when cultured with these liposomes containing RSL3 in all three cell lines (Supplementary Figure S7). These increases in lipid ROS were preventable with liproxostatin-1, indicative of ferroptosis (Supplementary Figure S7). The effects of the RSL3-encapsulated liposomes were not assessed against OPM-2 MM cells due to their sensitivity to RSL3 and because the RSL3 concentrations that would be required in liposomes for this cell line were below the limit of detection of the HPLC assay.

## 3. Discussion

Conventional cancer therapies, particularly chemotherapeutic regimens, typically induce tumour cell death by triggering apoptotic pathways. However, the significant proportion of cancer patients who are initially treatment-resistant or who relapse with drug-resistant disease highlights the pressing need for the development of new and improved therapeutic approaches.

Despite advances in the treatment of MM, it is still considered an incurable disease. Ferroptosis may represent a new avenue for treatment of this and other forms of cancer. However, it is apparent from the current study (Figure 1A) and that of Yang et al. [9], that MM cells are relatively insensitive to ferroptosis-mediated cell death induced either by direct (RSL3) or indirect (erastin) inhibition of GPX4, compared to DLBCL cells. The current study was undertaken to explore the role of PLs in enhancing the sensitivity of MM cells to ferroptosis and whether manipulation of the lipidome of these cells using novel liposomes represents an approach for harnessing this form of programmed cell death.

Unlike the other 5 MM lines investigated in this work, OPM-2 cells were significantly more sensitive to the GPX4 inhibitor, RSL3 (Figure 1A). This was apparent as cell death and characteristic morphological changes were induced by relatively low concentrations of the drug and both effects were inhibited by the synthetic antioxidant, liproxstatin-1. In comparison, IC_50_ values for RSL3 in the other MM lines were up to 95 times higher. Although we observed significant variation in GPX4 expression between the different lines (Figure 1B,C), no correlation between their sensitivity to RSL3 and expression of its target, GPX4, or trends in GPX4 expression between the MM and DLBCL lines, were apparent (Figure 1).

PLs play a pivotal role in ferroptosis, particularly lipids containing PUFA [12]. By performing a lipidomic analysis of MM and DLBCL cells, we showed that the MM cells studied generally contained a significantly lower proportion of PLs containing PUFAs, and a higher proportion of MUFA, than DLBCL cells (Figure 2A). The exception were the OPM-2 MM cells, which in terms of their proportions of PUFA and MUFA, were similar to DLBCL cells rather than the other MM lines (Figure 2B). Furthermore, OPM-2 cells, like DLBCL cells, were sensitive to RSL3 with IC_50_ values in a nanomolar range (Figure 1A). Overall, in the cell lines studied, the data demonstrated a strong link between the proportion of PUFA-containing PLs and the sensitivity of cells to RSL3.

The importance of the balance between PUFA and MUFA content is further highlighted by our finding that uptake of PUFAs is sufficient to induce ferroptosis (Figure 3B) and sensitise MM cells to RSL3 (Figure 4A). Our observation that OPM-2 and KMS-11 cells were significantly more sensitive to PE (16:0_22:6) than to PE (16:0_20:4) (Figure 3B,C), in terms of both ferroptosis-mediated cell death and lipid oxidation, is consistent with a previous study, suggesting that the degree of PL acyl chain saturation relates to ferroptosis sensitivity [12]. Oxidation of PUFAs during ferroptosis is known to result in their degradation, with a concomitant increase in levels of lysophospholipid species [9,45]. Accordingly, we observed an increase in the proportion of lysophospholipids in both OPM-2 and KMS-11 MM cells following culture with non-cytotoxic concentrations of PE (16:0_22:6) (Figure 3D). This finding indicates that even at low concentrations, addition of lipid substrates is sufficient to induce an increase in acyl chain oxidation and may prime normally ferroptosis-insensitive cells to this form of cell death. This is supported by the synergy observed between unsaturated PE lipids and RSL3 (Figure 4), where a non-cytotoxic dose of lipids significantly decreased the IC_50_ value for RSL3 in KMS-11 and OPM-2 MM cells. The effects of exogenous PUFAs on ferroptosis-resistant MM cells reinforce the notion that these cells do inherently express the machinery to undergo ferroptosis, but may lack sufficient endogenous ferroptosis substrates required to initiate this cell death process [31,32].

The contrasting effects of MUFA supplementation on the RSL3 sensitivity of OPM-2 cells (Figure 4B) further illustrates the strong association between fatty acid composition and the ferroptosis sensitivity of MM cells. Under basal conditions, OPM-2 cells had a significantly higher proportion of PUFAs than KMS-11 cells. The addition of exogenous MUFA to OPM-2 cells raised the intracellular proportion of this lipid and possibly displaced PUFAs from the lipidome, thereby significantly reducing the sensitivity of these cells to RSL3. Similar effects of MUFAs have been observed in human epithelial cells and mouse fibroblasts suggesting that the anti-ferroptotic effects of MUFAs are not limited to MM cells [38,50].

Iron is essential for cellular homeostasis [51], with key roles in oxygen transport, oxidative phosphorylation and DNA biosynthesis [52]. As iron chelation inhibits ferroptosis, this form of cell death is also clearly an iron-dependent process [8]. Intracellular iron levels are primarily regulated by the iron-storage protein ferritin, and the transferrin receptor (TfR) which shuttles transferrin-bound iron into the cell through receptor-mediated endocytosis. The level of non-protein bound iron (labile iron pool) has implications in ferroptosis as labile iron reacts with hydrogen peroxide inside cells, yielding highly reactive hydroxyl radicals in a process known as the Fenton reaction [53]. These radicals indiscriminately damage all surrounding organic material within a range of a few nanometres, resulting in cellular damage [53]. Iron also plays a role in ferroptosis through its actions on a group of iron-containing enzymes that mediate lipid peroxidation, known as lipoxygenases (LOXs) [19,54]. The key role of these enzymes is demonstrated by the LOX inhibitor, zileuton, which confers resistance to ferroptotic cell death in HT22 neuronal cells [55]. Furthermore, genetic knockdown or pharmacological inhibition of arachidonate lipoxygenases (ALOXs) protects cells against ferroptosis induced by erastin [56]. While labile iron and LOX expression or activity were not investigated in the current study, our focus on PLs remains mechanistically valid due to the observed dependence of RSL3-induced MM cell death on lipid ROS and prevention by ferroptosis inhibitors such as liproxstatin-1.

The ability to encapsulate drugs or to bind molecules, such as antibodies, to the surface of liposomes, means they represent an extremely versatile form of nanoparticles. We were able to show that liposomes manufactured from specific lipids, identified from the lipidomic screen of MM and DLBCL cells, were readily taken up by MM cells, leading to lipid ROS accumulation and ferroptosis-mediated cell death (Figure 5). This further demonstrates the crucial role of PUFAs as ferroptosis substrates and important determinants of MM cell sensitivity to this form of cell death. Moreover, this highlights the potential of ferroptosis-inducing liposomes as personalised or “precision” therapeutics, as it is conceivable that liposomes could be manufactured for specific patients, based on the lipidome of their tumour cells. As observed with exogenous PUFAs and free RSL3, liposomal delivery of PUFA combined with free RSL3 also exhibited a high degree of synergy against OPM-2 and KMS-11 MM cells, in terms of lipid ROS accumulation and ferroptosis-mediated cell death (Figure 5). While previous studies have used liposomes to deliver ferroptosis-inducing compounds to cancer cells, few have shown that the lipids used to manufacture the liposomes themselves are alone sufficient to induce ferroptosis or that this effect can be further enhanced by encapsulating RSL3 within the liposomes [23,24,25,26,27,28].

The current study demonstrated that it is possible to encapsulate RSL3 at concentrations of approximately 113 µM, a level that represented around 5% of the mass of the liposomes. Subsequent treatment of three MM cell lines showed that RSL3 delivered in this liposomal formulation was on average 7.1-fold to 14.5-fold more effective than free RSL3, represented by a decrease in the IC_50_s for cell viability from the micromolar to the nanomolar range (Figure 1A and Figure 6B). Furthermore, significantly fewer RSL3-encapsulated liposomes than empty liposomes were required to induce a similar degree of ferroptosis-mediated cell death (Figure 5B and Figure 6B). Future development of our liposomes will include external functionalisation with a monoclonal antibody against a MM-specific cell surface protein such as B cell maturation protein (BCMA), for tumour-directed cell death and sparing of healthy tissues, in preparation for in vivo testing. Such in vivo models include the syngeneic immunocompetent model utilising murine MM 5TGM1 cells in C57BL/KaLwRij mice [34] and human MM xenografts using NOD scid gamma (NSG) mice [57]. Finally, the challenges of translating liposome therapeutics for in vivo use are substantial and include stability, clearance, opsonisation and deactivation, and off-target effects [58]. Some of these have been considered in our ex vivo developmental process including liposome size and charge, inclusion of DSPE-PEG2000 and external functionalisation with a tumour-specific monoclonal antibody; however, until such in vivo experimentation is undertaken, it is difficult to predict how successful our liposomes will be, despite their encouraging in vitro efficacy presented herein.

## 4. Methods

### 4.1. Drugs, Chemicals, and Other Reagents

(1S,3R)-RSL3 (RSL3), bortezomib, liproxstatin-1, Z-VAD-FMK and necrostatin-1s were purchased from Selleck Chemicals (Houston, TX, USA). The lipids, 16:0 PE, 16:0-18:1 PE, 16:0-18:2 PE, 16:0-20:4 PE and 16:0-22:6 PE were purchased from Sigma-Aldrich (St. Louis, MO, USA). FITC-conjugated Annexin V and Annexin V Binding Buffer were purchased from Becton Dickinson Biosciences (Franklin Lakes, NJ, USA). Propidium iodide was purchased from Sigma-Aldrich. BODIPY^TM^ 581/591 C11 (D3861) was purchased from Thermo Fisher Scientific (Waltham, MA, USA). Anti-GPX4 and anti-RIP3 rabbit monoclonal antibodies were purchased from Cell Signalling Technology (Danvers, MA, USA). Anti-actin mouse monoclonal antibody was purchased from Millipore (Burlington, MA, USA). Peroxidase-conjugated, goat anti-rabbit and goat anti-mouse secondary antibodies were purchased from Invitrogen (Waltham, MA, USA).

### 4.2. Cell Culture

The human MM cell line, RPMI-8226 (ATC CCL-155), was purchased from the American Type Culture Collection (ATCC, Manassas, VA, USA). The KMS-11 (JCRB1179) human MM cell line was purchased from CellBank Australia (Sydney, Australia). The LP-1 (ACC 41) and OPM-2 (ACC 50) human MM cell lines were purchased from the Leibniz Institute DSMZ—German Collection of Microorganisms and Cell Cultures GmbH (Braunschweig, Germany). The NCI-H929 line was kindly provided by Prof. Andrew Spencer (Monash University, Melbourne, VIC, Australia), and KMS-18 cells were kindly provided by Prof. Junia Melo (South Australia Pathology, Adelaide, Australia). The DLBCL cell lines SU-DHL-8, OCI-Ly19, Farage, U-2932 and HBL-1 were supplied by Dr Giles Best (Adelaide, Australia). All cells were cultured in RPMI-1640 (Gibco, Waltham, MA, USA) supplemented with 10% foetal bovine serum, 50 units/mL penicillin, 0.25 mg/mL streptomycin, 2 mM L-glutamine and 15 mM HEPES buffer (all Gibco). Cells were maintained at 37 °C in 5% CO_2_. All cell lines were genetically authenticated by the Australian Genome Research Facility (AGRF; Adelaide, Australia) and determined mycoplasma-free using the MycoStrip^TM^—Mycoplasma Detection Kit (InvivoGen, San Diego, CA, USA). For cell culture, viable cells were enumerated by mixing 10 µL of cell suspension in a 1:1 ratio with 0.4% trypan blue (Invitrogen) and counted using a haemocytometer (Adelab, Adelaide, Australia).

### 4.3. Assessment of Cell Viability

Cells were initially cultured at a cell density of 3 × 10^5^ cells/mL with or without treatment for up to 24 h. Cells were washed in phosphate-buffered saline (PBS, Gibco, Waltham, MA, USA) and stained with 0.27 μg/mL FITC-conjugated Annexin V and 0.4 μg/mL propidium iodide (PI) in 1X Annexin V Binding Buffer for 10 min in the dark at room temperature. Intact cells were gated based on their size (forward scatter, FSC) and internal complexity (side scatter, SSC). Doublets were excluded by area scaling of the FSC area and height properties. Data from a minimum of 10,000 intact single cells was acquired either on a CytoFLEX S or CytoFlex SRT flow cytometer (Beckman Coulter, Brea, CA, USA), with analysis performed using CytExpert Software v2.4 or CytExpert SRT Software v1.0 (Beckman Coulter), respectively. Cells negative for both Annexin V and propidium iodide were considered viable. For cell death inhibition experiments, cells were preincubated in 200 µM Z-VAD-FMK for 45 min to inhibit apoptosis prior to the addition of RSL3 or bortezomib whereas 2 µM liproxstatin-1 to inhibit ferroptosis or 1 µM necrostatin-1s to inhibit necroptosis were added at the same time, prior to assessing cell viability by flow cytometry.

### 4.4. Assessment of Lipid ROS

Cells were cultured with or without treatment for 24 h at 37 °C. 30 min prior to the end of the incubation, a final concentration of 400 nM C11 BODIPY-FITC was added to relevant wells before a further 15 min incubation. Cells were then washed twice with PBS and re-suspended in fresh PBS prior to analysis by flow cytometry, as described above.

### 4.5. Sample Preparation for Lipidomic Analyses

Cells were cultured at a density of 3 × 10^5^ cells/mL with or without treatment for 4 h. Cell suspensions were washed in PBS and stored at −80 °C before processing. Subsequent sample processing and analysis by liquid chromatography/mass spectrometry was performed in the Lipidomics and Metabolomics core facility at the South Australian Health and Medical Research Institute (SAHMRI, Adelaide, Australia). All samples were prepared in duplicate and protein concentrations determined using the bicinchoninic acid (BCA) assay (Thermo Fisher Scientific), as per the manufacturer’s instructions. An extraction buffer consisting of acetonitrile/isopropanol/splash mix (99:99:2, *v*/*v*) was made fresh and 100 μL added to the equivalent of 10 μg of protein from each sample. Samples were then sonicated for 10 min and incubated at −20 °C for one hour. Samples were centrifuged at 16,000× *g* for 15 min and the supernatant transferred to a glass vial. A pooled quality control sample was prepared by combining 10 μL of supernatant from all samples in a glass vial. Samples were then run on a XEVO G2-XS QTOF liquid chromatography/mass spectrometer (Waters Corporation, Milford, MA, USA) according to a lipidomics assay protocol established at SAHMRI [59]. Sample data were processed and analysed using Skyline Targeted Mass Spec Environment v23.1 [60], MetaboAnalyst v5.0 and v6.0 (Wishart Research Group, Alberta, Canada) and Microsoft Excel v16.96. Heatmaps and Volcano plots were generated using MetaboAnalyst. Data were normalised to the median, log_10_ transformed, auto-scaled (mean-centred) and divided by the standard deviation of each variable.

### 4.6. Live Cell Imaging

50 μL poly-L-ornithine (Sigma-Aldrich) was added to each well of a 96 well plate and incubated for 1 h at room temperature. Excess solution was then removed from the wells and the plates were dried for 1 h. 5000–10,000 cells, with or without treatment, were added to each well. Images were obtained every hour for up to 24 h using an IncuCyte^®^ S3 Live-Cell Analysis System and software v2023A (Sartorius Australia, Victoria, Australia) at 20× magnification.

### 4.7. Western Blotting

3 to 5 million cells were lysed in 10 mM Tris/HCl (pH 7.4), 137 mM NaCl containing 10% glycerol, 1% NP40, 10 mM β-glycerophosphate, 2 mM sodium fluoride and complete EDTA-free protease inhibitor cocktail (Sigma-Aldrich). The samples were kept on ice for 10 min before centrifugation at 14,000× *g* to clarify the lysate. Equal amounts of protein were loaded onto a Mini-PROTEAN TGX Precast Gel (Bio-Rad, Hercules, CA, USA) and transferred onto nitrocellulose membranes using a Trans-Blot Turbo Transfer System (Bio-Rad). Membranes were then blocked in 5% non-fat dairy milk powder in 50 mM Tris (pH 7.4), 154 mM NaCl and 1% Tween20 at room temperature for two hours. Primary and secondary antibodies were used as per the manufacturers’ instructions and membranes were imaged on a ChemiDoc MP system (Bio-Rad) after incubation in Clarity Western ECL Substrate (Bio-Rad). GPX4, RIP3 and anti-actin primary antibodies were used at a dilution of 1:1000, 1:1000 and 1:5000, respectively, and HRP-conjugated secondary antibodies were used at a dilution of 1:15,000. Image Lab Software v6.1 (Bio-Rad) was used for densitometric analysis of Western blot images.

### 4.8. Liposome Preparation

#### 4.8.1. Micro-Fluidics Synthesis

A NanoAssemblr^®^ Ignite system (Precision Nanosystems, Vancouver, BC, Canada) was used to prepare all formulations using the following parameters: total flow rate = 12 mL/min, flow ratio = 3:1 (aqueous: organic), total volume = 4mL, start and end waste = 0.01 mL. Lipids were dissolved in 1mL of ethanol as the organic phase to produce a final lipid concentration of 1 mg/mL. Drug free liposomes were prepared using PBS (pH = 7.4) as the aqueous phase. Liposomes were prepared in an organic phase consisting of PE (16:0_22:6): DSPE-PEG2000 at ratios of 98:2 (*w*/*w*%). Synthesised liposomes were dried under N_2_ gas to remove excess solvent. Liposomes containing a Rhodamine B-conjugated lipid at a concentration of 1 μg/mL were also synthesised to enable liposome uptake to be assessed by flow cytometry.

#### 4.8.2. Liposome Characterisation

The various liposome formulations were characterised by dynamic light scattering (DLS) using a Malvern Zetasizer Nano ZS at 25 °C and Zetasizer Nano software v3.30 (Malvern Panalytical, Worcestershire, UK). Samples were diluted 10-fold with Milli-Q ultrapure water (Merck Millipore, Burlington, MA, USA) for all size measurements. Results were reported as a mean hydrodynamic diameter ± standard deviation, and polydispersity index (PDI). Zeta potential was also measured using the Malvern Zetasizer. Undiluted samples were used for zeta potential measurements, with results reported as the average zeta potential ± standard deviation.

#### 4.8.3. Assessment of Liposome Uptake

Cells were cultured, with or without, liposomes containing the PE (18:1) (lissamine rhodamine B sulfonyl)-conjugated lipid (approximately 1 μg/mL in 1 mg/mL liposome concentration) at concentrations of up to 50 μg/mL, for 24 h at 37 °C. Cells were then washed with PBS and resuspended in fresh PBS. The proportion of cells containing the fluorochrome-tagged lipid was determined by flow cytometry, as described above.

#### 4.8.4. Assessment of Liposome RSL3 Encapsulation

Liposomes were prepared, with or without, varying concentrations of RSL3. Ultrafiltration using an Ultracell Ultrafiltration system fitted with a PES 5 kDa membrane filter (Merk Millipore, Burlington, MA, USA) was then used to remove free RSL3. The liposome solutions were weighed before and after filtration to account for volume lost during filtration before dilution at a 1:1 ratio in the mobile phase and analysis as described below.

#### 4.8.5. High-Performance Liquid Chromatography

High-performance liquid chromatography (HPLC—Shimadzu Nexera XR) and a Phenomenex Luna 5 µm C18(2) 100 Å, LC Column 250 × 4.6 mm (Phenomenex, Torrance, CA, USA) HPLC column were used to determine the concentration of RSL3 encapsulated within the liposomes. Conditions were as per manufacturer’s instructions and are described below, with alterations to account for equipment variability. The column oven was set to 25 °C, with a wavelength of 280 nm and flow rate of 0.8 mL/min. Mobile phase A consisted of H_2_O and 0.1% trifluoroacetic acid (TFA), mobile phase B was composed of acetonitrile and 0.1% TFA. Naphthalene was used as an internal standard at a concentration of 100 µg/mL. The injection volume was 10 µL and the isocratic method used was 10% mobile phase A/90% mobile phase B. RSL3 standards, ranging from 5 µg/mL–250 µg/mL, and unknown samples were prepared in mobile phase B. Peaks were detected at a retention time of 1.9 min. All samples were measured in triplicate with a minimum of three biological replicates.

### 4.9. Statistical Analyses

Statistical analyses were performed by Student’s *t*-test for two-group comparisons and two-way ANOVA for comparing more than two groups, using GraphPad Prism software v10.2.0 (Boston, MA, USA). A *p*-value < 0.05 was considered statistically significant, with differing degrees of statistical significance indicated as follows: * *p* < 0.05, ** *p* < 0.01, *** *p* < 0.001, **** *p* < 0.0001. The fractional product method was used to determine synergistic, additive or antagonistic effects of drug combinations, with values of <0.1 indicative of synergy [47]. GraphPad Prism was used to fit 4-parameter logistic (4PL) sigmoidal dose–response models for cell viability data to determine IC_50_ values.

## 5. Conclusions

Collectively, the results of this study demonstrate a significant link between the polyunsaturated and monounsaturated fatty acid composition of MM cells and their sensitivity to ferroptosis. By manipulating the intracellular PL composition, we demonstrate that it is possible to markedly sensitise MM cells to ferroptosis induced by GPX4 inhibition. Our liposomes manufactured from PL-PUFAs that are relatively deficient in MM cells and loaded with RSL3 provide strong proof-of-principle evidence that lipid nanoparticles may represent a highly effective mechanism for delivering both ferroptosis substrates and ferroptosis-inducing agents to cancer cells. Moreover, by manufacturing liposomes with specific PL-PUFAs that are less abundant in MM cells compared to ferroptosis-sensitive cancers, and by extension, less abundant in MM cells from a given MM patient compared to another MM patient, personalised or precision ferroptosis-inducing liposomes could be developed. These findings support further research and development of therapeutics that induce ferroptosis as a novel treatment approach for MM and other forms of cancer, and particularly for overcoming resistance to more conventional apoptosis-inducing therapies.

## Data Availability

The datasets used and/or analysed during the current study are available from the corresponding author on reasonable request.

## Supplementary Materials

The following supporting information can be downloaded at: https://www.mdpi.com/article/10.3390/ijms26146579/s1.

## List of Abbreviations

DLBCL	Diffuse large B cell lymphoma
FA	Fatty acid
GPX4	Glutathione peroxidase 4
HPLC	High-performance liquid chromatography
LC-MS	Liquid chromatography–mass spectrometry
LIP-1	Liproxstatin-1
LOX	Lipooxygenase
LPC	Lysophosphatidylcholine
LPE	Lysophosphatidylethanolamine
MM	Multiple myeloma
MUFA	Monounsaturated fatty acid
NEC-1	Necrostatin-1
PC	Phosphatidylcholine
PCD	Programmed cell death
PDI	Polydispersity index
PE	Phosphatidylethanolamine
PI	Propidium iodide
PL	Phospholipid
PS	Phosphatidylserine
PUFA	Polyunsaturated fatty acid
RIPK	Receptor-interacting protein kinase
ROS	Reactive oxygen species
RSL3	(1S,3R)-RSL3
SFA	Saturated fatty acid
