# Supplementary material for: Novel (1S,3R)-RSL3-Encapsulated Polyunsaturated Fatty Acid Rich Liposomes Sensitise Multiple Myeloma Cells to Ferroptosis-Mediated Cell Death
Source: Int J Mol Sci. 2025 Jul 9;26(14):6579. doi: 10.3390/ijms26146579 (PMC12294587; doi:10.3390/ijms26146579)
Supplement: Supplementary file 1 [file ijms-26-06579-s001.zip › ijms-3712603-supplementary.pdf]

Additional files

File name:

Supplementary File S1

File format:

.mpv (movie file)

Title of data:

N/A

Description of data:

OPM-2 cells were cultured with PE (16:0_22:6) and images captured over a 24-hour time frame using an IncuCyte S3 instrument. The images show that the cells underwent morphological changes characteristic of ferroptosis, including cytoplasmic "ballooning", in response to addition of the lipid. This movie shows the cellular morphology changes up to 12 hours.

Supplementary figures

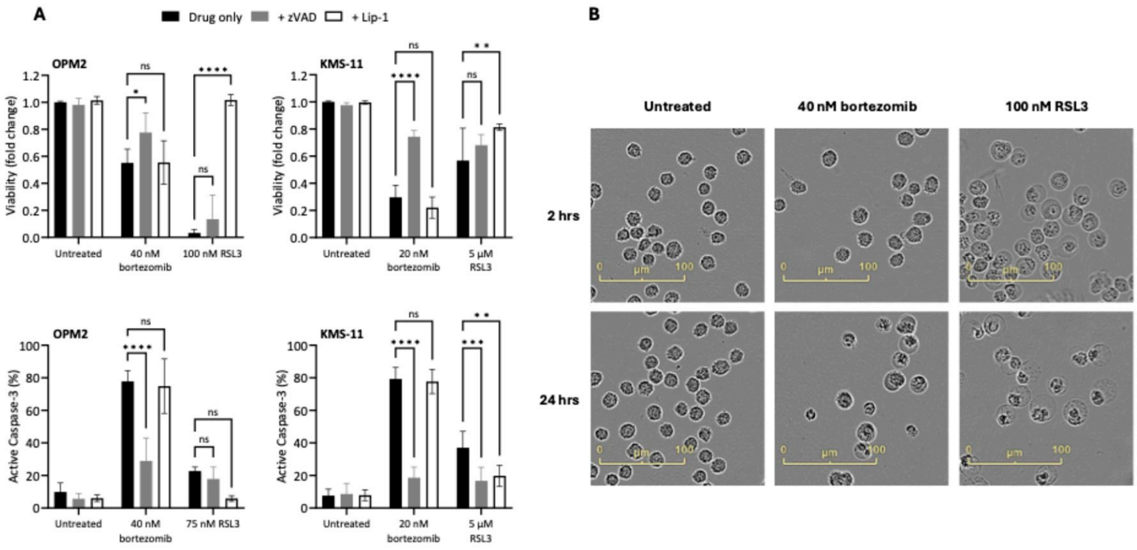

Supplementary Figure S1. Cell death induced by RSL3 is preventable with liproxstatin-1, but not Z-VAD-FMK.

KMS-11 and OPM2 cells were treated with the indicated concentrations of bortezomib or RSL3 for 24 hours. For cell death inhibition, cells were preincubated in 200 μM Z-VAD-FMK for 45 minutes whereas 2 μM liproxstatin-1 (Lip-1) was added at the same time as bortezomib/RSL3. (A) Cell viability was measured by flow cytometry. Data are the mean ± standard deviation from three independent experiments. Statistical analyses were performed by two-way ANOVA with a Tukey's multiple comparisons test; ns indicates no significant difference, *$p < 0.05$, **$p < 0.01$, **** $p < 0.0001$. (B) OPM2 cells were treated with bortezomib or RSL3 with images acquired over 24 hours using an IncuCyte S3 live cell analysis system at 20x magnification.

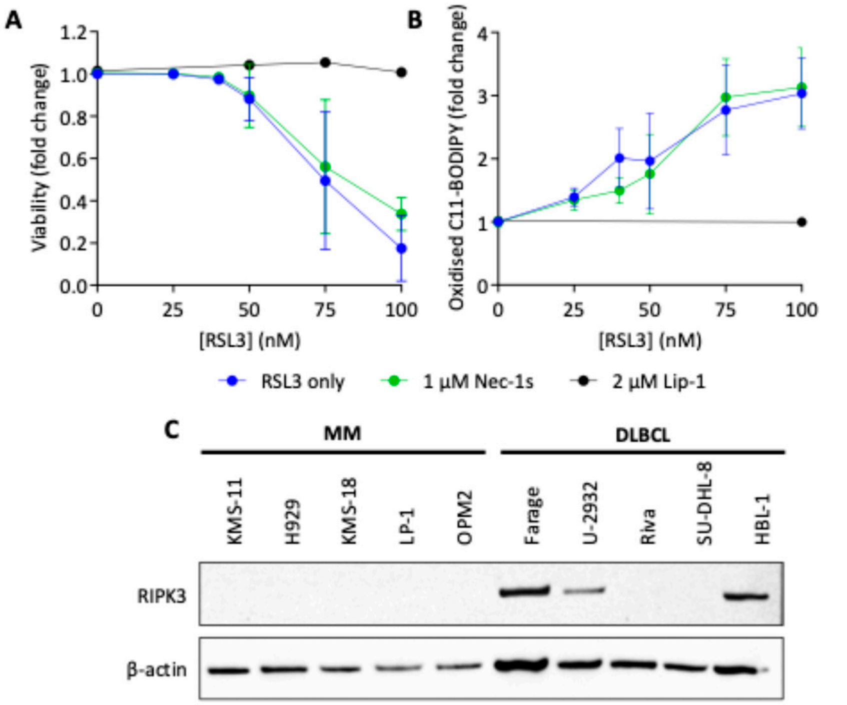

Supplementary Figure S2. RSL3-induced death is not dependent on RIPK3 in MM cells.

OPM2 cells were treated with the indicated concentrations of RSL3 ± 2 µM Liproxstatin-1 (Lip-1) or 1 µM Necrostatin-1s (Nec-1s) for 24 hours. Cell viability (A) and the levels of oxidised C11-BODIPY (B) were measured by flow cytometry. Data are the mean ± standard deviation from three independent experiments. (C) Receptor-interacting protein kinase 3 (RIPK3) protein levels were assessed in untreated MM and DLBCL cells by Western blotting, with β-actin used as a loading control. MM, multiple myeloma; DLBCL, diffuse large B cell lymphoma.

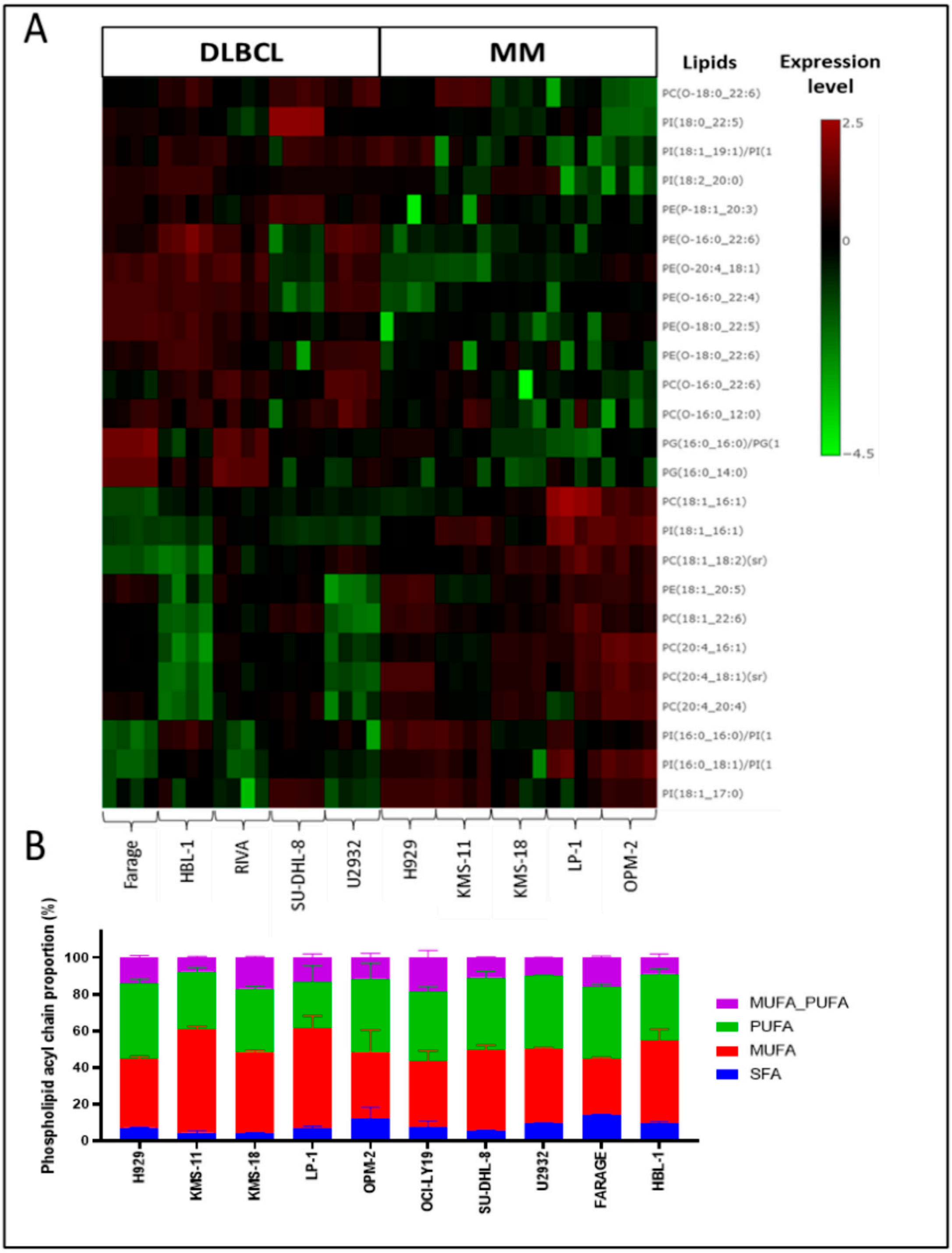

Supplementary Figure S3. Fatty acid expression patterns in MM and DLBCL cell lines.

(A) Lipidomics analysis of 5 DLBCL (Farage, HBL-1, RIVA, SU-DHL-8, U2932) and 5 MM (H929, KMS-11, KMS-18; LP-1, OPM-2) cell lines. Heatmap of the 25 most differentially expressed lipids between the MM and DLBCL lines. **(B)** Relative proportions of each PL acyl chain present in the MM and DLBCL cell lines. Data are the mean of a minimum of 4 biological replicates. MM, multiple myeloma; DLBCL, diffuse large B cell lymphoma; PC, phosphatidylcholine; PE, phosphatidylethanolamine; PG, phosphatidylglycerol; PI, phosphatidylinositol; MUFA, monounsaturated fatty acid; PUFA, polyunsaturated fatty acid; SFA, saturated fatty acid.

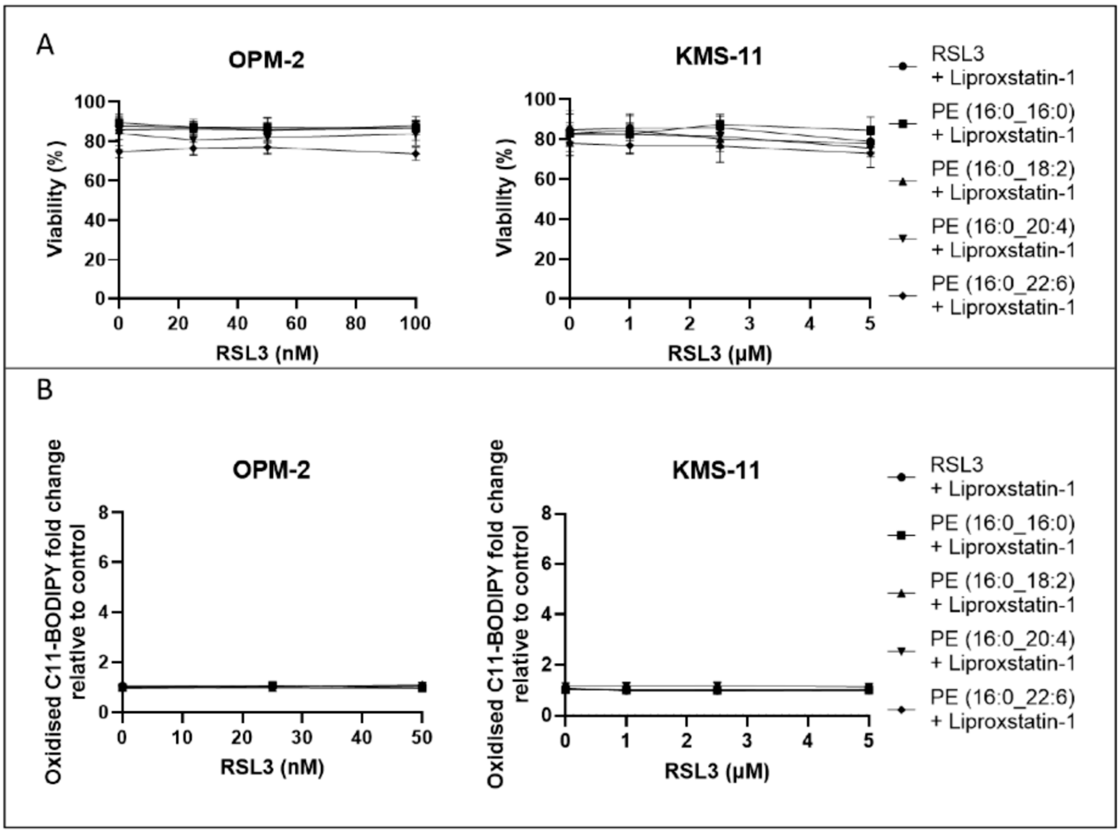

Supplementary Figure S4. Liproxstatin-1 inhibits cell death and lipid ROS induced by PUFA and RSL3.

(A) OPM-2 and KMS-11 cells were cultured with 20 µM PE lipids and the indicated concentrations of RSL3 with liproxstatin-1 for 24 hours. Cell viability was assessed using annexin V/PI staining and flow cytometry. **(B)** OPM-2 and KMS-11 cells were cultured with 20 µM PE lipids and the indicated concentrations of RSL3 with liproxstatin-1 for 24 hours. Lipid ROS levels were assessed by flow cytometry in cells stained with oxidised C11 BODIPY. Data are the mean ± standard deviation of duplicate measurements from 3 independent experiments. PE, phosphatidylethanolamine.

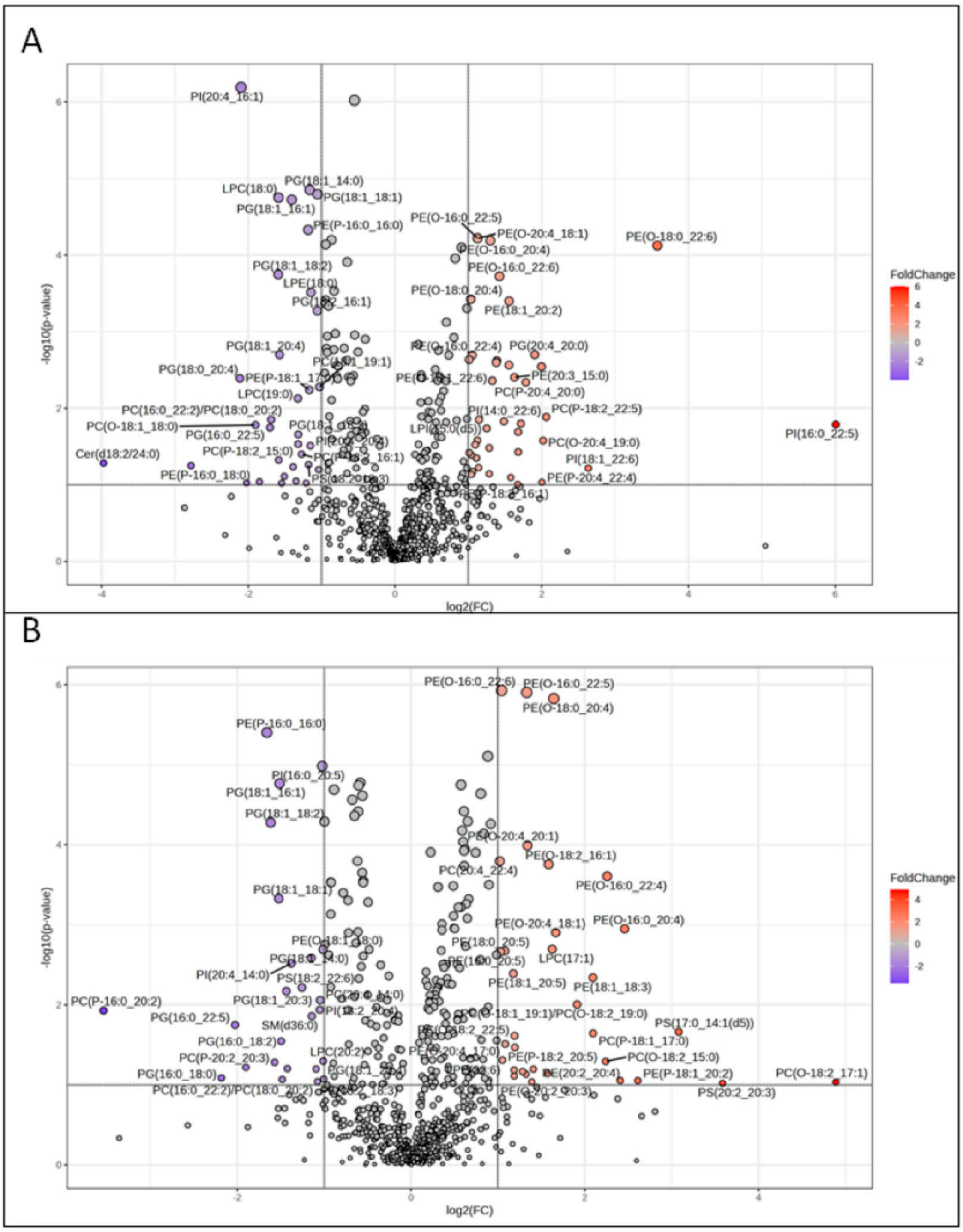

Supplementary Figure S5. Oleic acid (18:1) displaces PUFA in phospholipids in OPM-2 and KMS-11 cells.

(A) Volcano plot (direction of comparison OPM-2/OPM-2 + PE (16:0_18:1)) combining results from Fold Change (Threshold 2.0) Analysis and t-tests (P-value threshold 0.1) generated using MetaboAnalyst. **(B)** Volcano plot (direction of comparison KMS-11/KMS-11 + PE (16:0_18:1)) combining results from Fold Change (Threshold 2.0) Analysis and t-tests (P-value threshold 0.1) generated using MetaboAnalyst. Data are the mean of a minimum of 4 biological replicates. LPC, lysophosphatidylcholine; PC, phosphatidylcholine; PE, phosphatidylethanolamine; PG, phosphatidylglycerol; PI, phosphatidylinositol. PS, phosphatidylserine.

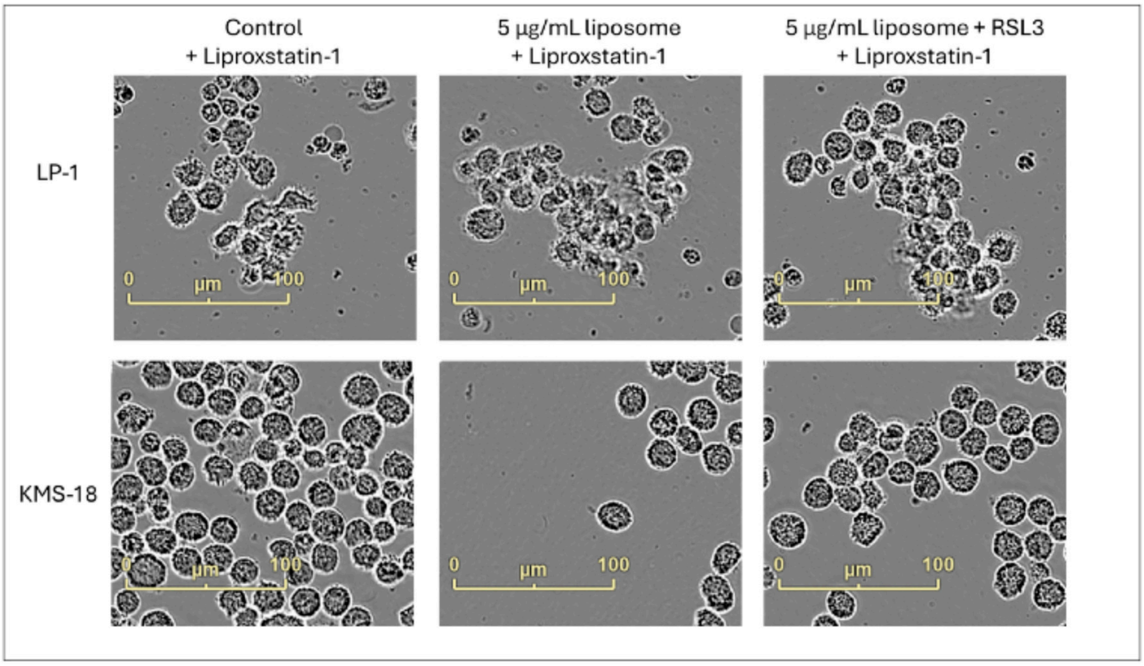

Supplementary Figure S6. Liproxstatin-1 inhibits morphological changes induced by liposomes encapsulating RSL3.

MM cells were cultured for 24 hours with 5 μg/mL liposomes that either did or did not contain RSL3, in addition to liproxstatin-1. Images were acquired at 24 hours using an IncuCyte S3 live cell analysis system at 20x magnification.

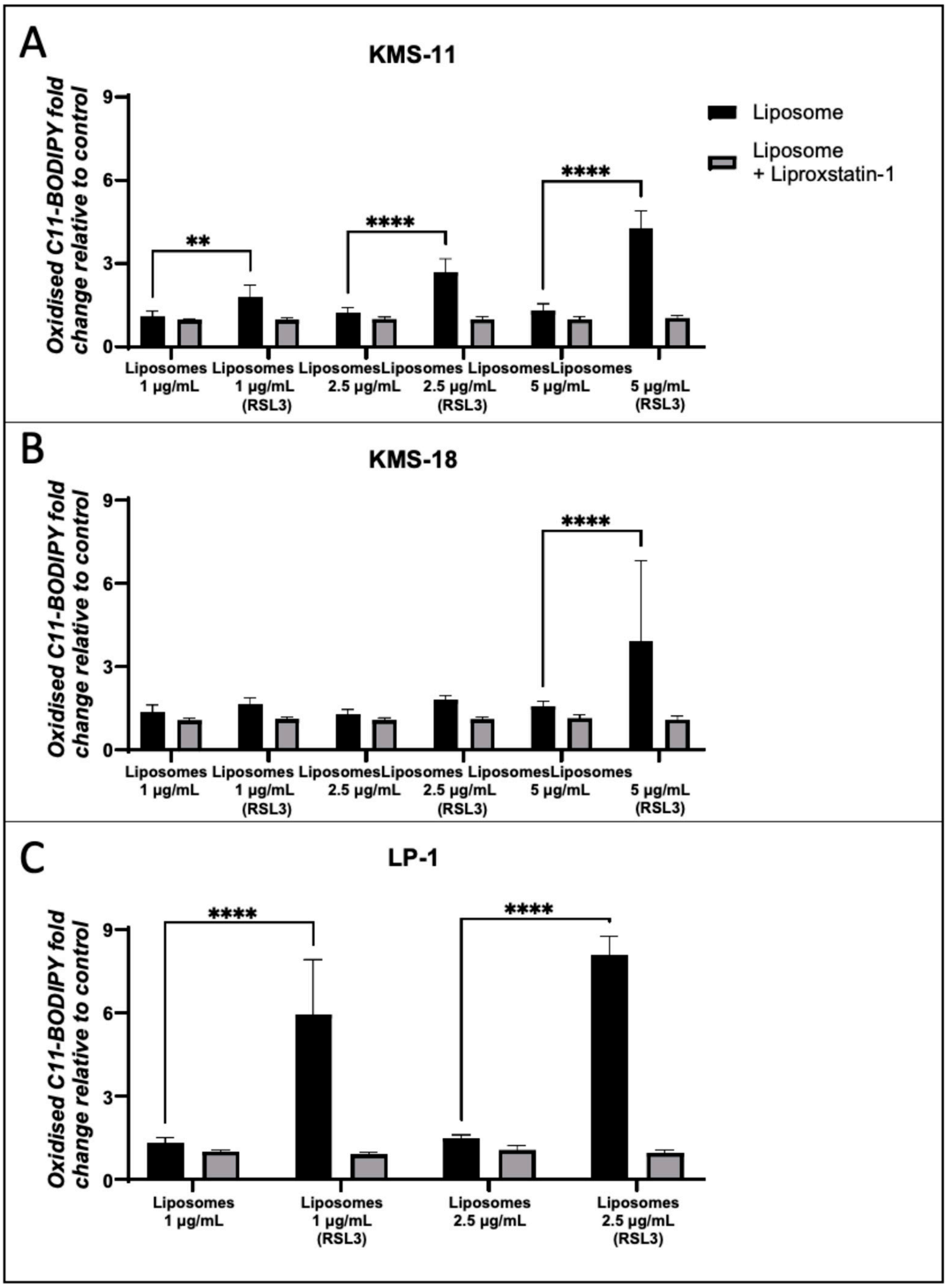

Supplementary Figure S7. Liposomes containing RSL3 induce greater lipid ROS in MM cell lines compared to liposomes alone.

(A) KMS-11, **(B)** KMS-18 and **(C)** LP-1 MM cells were cultured with the indicated concentrations of either liposomes alone or liposomes containing RSL3 for 24 hours. Lipid ROS levels were assessed by flow cytometry in cells stained with C11 BODIPY. Data are the mean ± standard deviation of duplicate measurements from 3 independent experiments using two-way ANOVA for statistical analysis (**** $p < 0.0001$).
